# Characterization and adsorption properties of cross-linked yeast/β-cyclodextrin polymers for Pb(ii) and Cd(ii) adsorption

**DOI:** 10.1039/c8ra06171h

**Published:** 2018-09-10

**Authors:** Zhengyang Duan, Mingyao Song, Tianguo Li, Shuli Liu, Xiaojun Xu, Ronggao Qin, Changhua He, Yao Wang, Longqian Xu, Mengjiao Zhang

**Affiliations:** Faculty of Environmental Science and Engineering, Kunming University of Science and Technology Kunming 650500 PR China; College of Resources and Environment, Yunnan Agricultural University Kunming 650201 PR China; Faculty of Land Resource Engineering of Kunming University of Science and Technology Kunming 650500 PR China rgqinkmust@sina.com +86-18213456773

## Abstract

In this study, a crosslinked yeast/β-cyclodextrin polymer (Y–β-CDP), for use as an effective adsorbent for removal Pb(ii) and Cd(ii) ions from aqueous solution, has been innovatively prepared by grafting β-cyclodextrin (β-CD) onto the surface of baker's yeast (BY) and thiomalic acid as a crosslinker. Several characterization techniques, such as SEM equipped with an EDS analyzer, FTIR, XRD, and XPS were employed characterize the Y–β-CDP. The impact of various operating parameters, such as pH, adsorbent dosage, initial concentration of metal ions, contact time and solution temperature, as well as adsorption kinetics, isotherms and thermodynamics were systematically investigated. The adsorption of Pb(ii) and Cd(ii) on Y–β-CDP reached equilibrium in 25 min, and the kinetic process conforms to the pseudo-second order model. The Langmuir model was used to describe the adsorption isotherm data better than the Freundlich model. The predicted maximum adsorption capacity at 25 °C for Pb(ii) and Cd(ii) was 150.08 and 102.80 mg g^−1^, respectively, when the initial concentration of metal ions was 120 mg L^−1^. The thermodynamic analysis revealed that the adsorption procedure of Pb(ii) and Cd(ii) onto Y–β-CDP was spontaneous and endothermic. Furthermore, regeneration experiments demonstrated that Y–β-CDP had excellent recyclability. Together, all results suggested that Y–β-CDP could potentially be a promising adsorbent in the purification of water contaminated with heavy metal ions.

## Abstract 2

A cross-linked yeast/β-cyclodextrin polymer (Y–β-CDP) was synthesized to remove Pb(ii) and Cd(ii) from aqueous solution.

## Graphical abstract

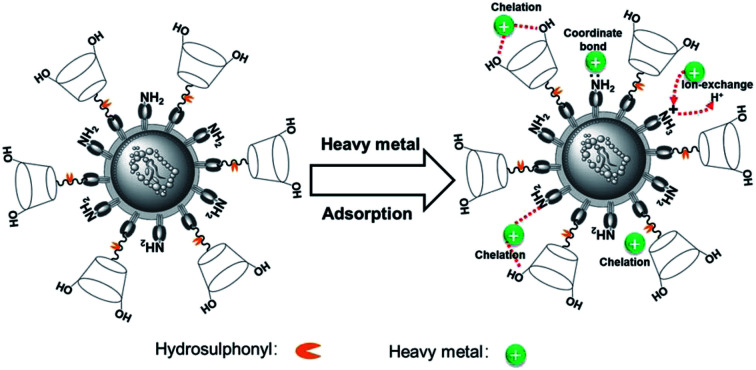

## 1. Introduction

Continuous industrial development and urbanization have been accompanied by a large amount of wastewater containing heavy metals being discharged into the environment. Heavy metal ions present in the environment are not only highly toxic, showing difficulty biodegrading or being eliminated by the natural environmental process, but can also be transported and enriched through the food chain, ultimately endangering human health.^1^ It is noteworthy that, similar to Hg(ii), Cr(iv), and As(iii), Pb(ii) and Cd(ii) have been classified as class I pollutants in industrial wastewater by the Ministry of Ecology and Environment of China.^2^ Similarly, the United States Environmental Protection Agency (USEPA) stipulates that the permissible limit of Pb(ii) and Cd(ii) in drinking water is 0.005 mg L^−1^ and 0.015 mg L^−1^, respectively.^3^ The lead (Pb) and cadmium (Cd) that are present for long periods of time in wastewater are generally considered to be involved in the development of cardiovascular disease, kidney damage, bronchiolitis, osteoporosis, and even certain types of cancers.^4,5^ Therefore, it is necessary to effectively purify the effluents containing Pb(ii) and Cd(ii) before being released into the environment.

Various methods, such as ion exchange, electrochemistry, chemical precipitation, adsorption, and membrane separation, have been used to remove Pb(ii) and Cd(ii) from aqueous solutions.^6–10^ In such methods, adsorption was recognized as the most effective and applicable method for the removal of heavy metal ions from wastewater. Over the past few decades, researchers have developed a number of adsorptive materials for heavy metal wastewater treatment, such as zeolite,^11,12^ activated carbon,^13^ chitosan,^14^ humic acid,^15^ and sawdust.^16^ Unfortunately, these adsorbents still have certain limitations when it comes to their practical application, such as low efficiency, high cost, sludge production, and poor reproducibility. Thus, the preparation of cost-effective adsorbents with high adsorption efficiency, no secondary pollution, and good reproducibility has become a demanding and challenging task.^17,18^

Baker's yeast (BY), an inexpensive, non-toxic, and readily available biological source that is often used to remove metal ions from wastewater taking advantage of the many functional groups on the yeast's cell wall, including hydroxyl, amino, and carboxyl groups.^19^ Nevertheless, the adsorption performance of natural BY is often unsatisfactory because of its low adsorption capacity and poor mechanical properties. It is hoped that the numerous functional groups found on the surface of BY have laid the foundation for its chemical modification. In an analogy, β-cyclodextrin (β-CD) is a cyclic oligosaccharide produced through the biodegradation of starch by enzymatic conversion, and it has attracted a considerable amount of attention due to the numerous hydroxyl groups present inside and outside of the β-CD molecule. These hydroxyl groups endow the molecule with the ability to capture pollutants, as well as to easily combine with various functional groups.^20,21^ However, its high water solubility limits its practical application in wastewater. At present, the crosslinking of BY and β-CD using an approach to form insoluble polymers that is both simple and environmentally friendly will be more suitable for the requirements of environmental protection. Other researchers have reported similar materials. Zhao and Repo have used trifunctional chitosan–EDTA–β-cyclodextrin polymer for the simultaneous removal of metals and organic micropollutants.^22^ He *et al.* used tetrafluoroterephthalonitrile (TFP) as a cross-linker to synthesize β-cyclodextrin polymers for the removal of Pb(ii), Cu(ii), and Cd(ii) from aqueous solutions.^23^ Recently, Wu *et al.* synthesized an EDTA modified β-cyclodextrin/chitosan for the removal of Pb(ii) and acid red from aqueous solution.^24^ Unfortunately, the materials mentioned above are either too complex to synthesize or the solvents/crosslinking agents are toxic. Therefore, it is necessary to find an environment-friendly cross-linking agent and green cross-linking technology to prepare yeast/β-cyclodextrin polymer adsorbent (Y–β-CDP).

**Scheme 1:**
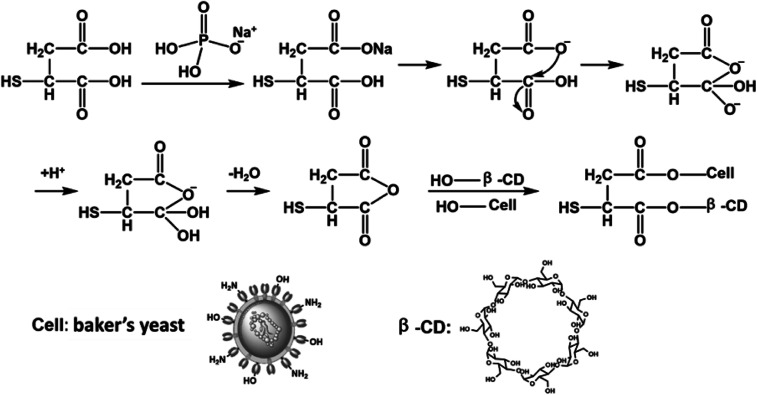
The synthetic diagram of Y–β-CDP.

In the above context, an adsorbent with low-cost and high removal efficiency has been prepared by grafting β-cyclodextrin (β-CD) onto the surface of baker's yeast (BY), using thiomalic acid as a crosslinker (Scheme 1). The work involves three benefits: (1) an environment-friendly cross-linking agent and green synthesis method were selected for the preparation of Y–β-CDP. (2) The crosslinking reaction was carried out with thiomalic acid instead of using glutaraldehyde (GLA), epichlorohydrin (EPI), tetrafluoroterephthalonitrile (TFP), and other toxic crosslinkers. (3) Thiomalic acid not only acts as a crosslinker, but its own sulfhydryl group also plays a key role in the combination of metal ions. The prepared Y–β-CDP was used as an adsorbent to evaluate the adsorption properties of Pb(ii) and Cd(ii) from aqueous solution. To optimize the experimental conditions, different experimental parameters, such as pH, dosage of adsorbents, contact time, initial concentration of Pb(ii) and Cd(ii) and temperature, were investigated. To further understand the behavior of adsorption, the isotherms, kinetics, and thermodynamics were used to analyze the experimental data. In addition, the physicochemical characteristics of the involved composite and the adsorption mechanism of Pb(ii) and Cd(ii) onto β-CDP were characterized by means of scanning electron microscope (SEM) and energy-dispersive X-ray spectroscopy (EDS), as well as Fourier transform infrared spectroscopy (FTIR), X-ray diffraction (XRD) and X-ray photoelectron spectroscopy (XPS) analyses.

## 2. Materials and methods

### 2.1 Materials and reagents

BY was purchased from Angel yeast Co., Ltd., (Hubei, China), before use, it was dried in an oven at 50 °C for 12 hours. Lead nitrate (Pb(NO_3_)_2_), cadmium nitrate (Cd(NO_3_)_2_·4H_2_O) and the catalyst sodium dihydrogen phosphate (NaH_2_PO_4_) were purchased from Sinopharm Chemical Reagent Co., Ltd., (Shanghai, China). β-CD (C_42_H_70_O_35_, 98%), thiomalic acid (C_4_H_6_O_4_S, 98%) and all other chemicals and reagents used in this study were supplied by Aladdin (China) and used as analytical reagent grade. Ultrapure water with a resistivity of 18.25 MΩ cm was used to prepare all the solutions in this study.

### 2.2 Preparation of Y–β-CDP

The synthesis of the Y–β-CDP was performed according to the theory that thiomalic acid cyclic anhydride occurs first under the sodium dihydrogen phosphate as catalyst, and then the BY was cross-linked with β-CD by esterification. Briefly, dried BY (2 g) and β-CD (5 g) were added into a 100 ml conical bottle containing 50 ml of deionized water and the mixture was evenly dispersed by stirring at 30 °C in an oil bath for 10 min. Then, thiomalic acid (2 g) and sodium dihydrogen phosphate (0.5 g) were added to the mixture, and the temperature was raised to 140 °C and stirring was continued for 35 min. After the reaction was completed, the obtained solids were repeatedly washed with deionized water and ethanol until the unreacted substances were removed. Eventually, the product was dried to a constant weight at 60 °C in a vacuum drying oven and used as adsorbent after grinding. The proposed synthetic processes is shown in Scheme 1.

### 2.3 Characteristics of Y–β-CDP

A Quanta 200 (SEM) microscope (FEI Co., Hillsboro, OR, USA) equipped with an EDS spectrometer was used to observe the surface morphology and analyze the chemical composition of the Y–β-CDP, before and after Pb(ii) and Cd(ii) adsorption. The changes of the functional groups on the BY, β-CD, and Y–β-CDP before and after Pb(ii) and Cd(ii) were identified by a FTIR spectroscopy using a Thermo Scientific Nicolet iS50 FTIR spectrometer (Thermo Fisher Scientific) in the range of 4000–400 cm^−1^ with KBr as a dispersant. A D/Max-2200 XRD diffractometer (Rigaku Corp., Tokyo, Japan) was used to analyze the phase composition of the samples. XPS analysis of Y–β-CDP before and after Pb(ii) and Cd(ii) adsorption were performed by using the ESCALAB250Xi X-ray photoelectron spectrometer (Thermo Fisher Scientific). The computer deconvolution of the XPS spectra was used to investigate the elements of the peaks of N 1s, O 1s, and S 2p in the Y–β-CDP. The zeta potential of Y–β-CDP was measured with a JS94H Zeta potentiometer (Yima Optoelec Co., Ltd., Xi'an, China).

### 2.4 Batch adsorption experiments

#### 2.4.1 Preparation of adsorbate solution

Stock solutions of 1000 mg L^−1^ of Pb(ii) or Cd(ii) were prepared by dissolving 1.6147 g of Pb(NO_3_)_2_ and 2.7714 g of Cd(NO_3_)_2_·4H_2_O in 1000 ml of ultra-pure water. Other concentrations required for the experiments were obtained by dilution of the stock solutions. The 0.1 mol L^−1^ NaOH and 0.1 mol L^−1^ HNO_3_ solutions were used to adjust the pH values required for the experiments.

#### 2.4.2 Adsorption experiments

Batch adsorption experiments were carried out by mixing Y–β-CDP with 100 ml of Pb(ii) and Cd(ii) solution in a 200 ml conical flask. The mixture was shaken at 120 rpm on a double thermostatic shaker. The effect of the pH on the adsorption capacity was determined in the pH range from 2.0–8.0, which was adjusted with dilute NaOH and HNO_3_ solutions at 25 °C. The influence of the dosage was evaluated by varying the dosage (5, 10, 20, 40, 60, 80, and 100 mg/100 ml) at pH 5 for Pb(ii) and pH 7 for Cd(ii) at the temperature of 25 °C.

The adsorption kinetics were evaluated by adding 20 mg of Y–β-CDP into a 200 ml conical flask containing 100 ml of metal ions solution at 25 °C. The initial concentration of metal ions in the solution was 25, 50 or 100 mg L^−1^ and the contact time of 1–50 min. The pH of the solution was adjusted to 5 for Pb(ii) and pH 7 for Cd(ii) and then shaken at 120 rpm.

The adsorption isotherms were measured in a 200 ml conical flask containing 100 ml of metal ions solution and 20 mg Y–β-CDP. The initial concentration of metal ions ranged from 10–120 mg L^−1^, the pH was adjusted to 5 for Pb(ii) and pH 7 for Cd(ii). The mixture was shaken at 120 rpm in a double thermostatic shaker at four different temperatures (25, 30, 35, and 40 °C) for 25 min.

Adsorption thermodynamics were investigated at the experimental temperatures range from 25–40 °C. Other experimental conditions comply with the above parameters.

After adsorption equilibrium, the supernatant solution was filtered using a 0.45 mm nylon sterile filter, and the residual concentration of Pb(ii) and Cd(ii) were determined with an atomic absorption spectrometer (AAS) (AA6300, Shimadzu, Kyoto, Japan). All experimental values were averaged after three measurements and the deviation was within ±5%. The adsorption capacity (*q*_e_) and removal rate (*η*) were calculated using the following two equations:^1^1
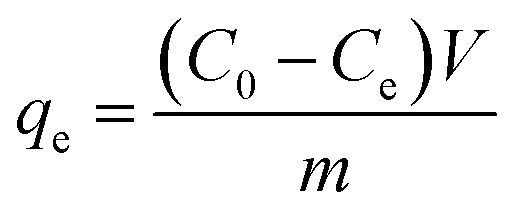
2
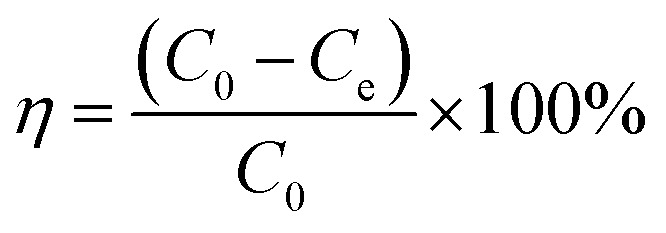
where *C*_0_ (mg L^−1^) and *C*_e_ (mg L^−1^) are the concentration of adsorbates in the initial and adsorption equilibrium, respectively; *V* (L) is the volume of the adsorbed solution and *m* (g) is the amount of adsorbent.

### 2.5 Regeneration experiment

In order to evaluate the regenerative properties of Y–β-CDP, 0.1 M HCl was used as desorption solution. In this process, 0.02 g of the Y–β-CDP was added to the solution containing metal ions (100 ml, 100 mg L^−1^). After the adsorption was saturated, the Y–β-CDP loaded metal ions were recovered and dispersed in 20 ml HCl solution at 150 rpm for 120 min to be desorbed. Subsequently, the Y–β-CDP was successfully recovered and dried at 40 °C for the next adsorption cycle experiment under the same conditions. After every cycle, the concentration of Pb(ii) and Cd(ii) in the supernatant was determined by AAS, as described above and then the adsorption capacity was obtained.

## 3. Results and discussion

### 3.1 Physicochemical properties of Y–β-CDP

#### 3.1.1 SEM and EDS analysis

**Fig. 1:**
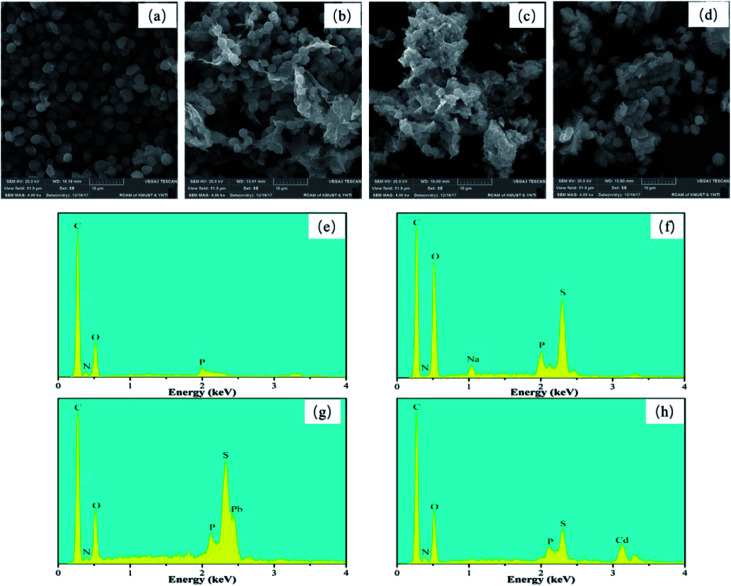
SEM images and EDS spectra of BY (a and e), Y–β-CDP (b and f), Y–β-CDP–Pb(ii) (c and g) and Y–β-CDP–Cd(ii) (d and h).

The SEM images displayed in Fig. 1a–d show the morphology of the samples surface. The images reveal that: (a) the pristine BY is ellipsoidal with smooth surface and uniform size (2.2 μm × 1.7 μm). (b) The Y–β-CDP became more rough and a network structure was formed due to the chemical crosslinking processes that had occurred on the surface. (c and d) A remarkable change occurred in the Y–β-CDP surface morphology, after Y–β-CDP was loaded with Pb(ii) or Cd(ii), indicating that metal ions were adsorbed on the Y–β-CD surface.

Similarly, the EDS spectra of BY, Y–β-CDP, and Y–β-CDP–Pb(ii)/Cd(ii) surfaces were obtained and are shown in Fig. 1e–h. The EDS spectra reveal that: (e) the main chemical components of BY were C, N, O, and P, as the primary components of BY are dextran, mannose, and chitin.^25^ (f) The S element appeared on the Y–β-CDP surface, indicating that the β-CD was successfully crosslinked to BY surface. (g and h) The EDS spectra of the Y–β-CDP–Pb(ii) and Y–β-CDP–Cd(ii) shown in Fig. 1g and h, respectively, reveal the presence of Pb and Cd peaks further confirming that Pb(ii) and Cd(ii) were adsorbed onto the Y–β-CDP polymer.

#### 3.1.2 XRD analysis

**Fig. 2:**
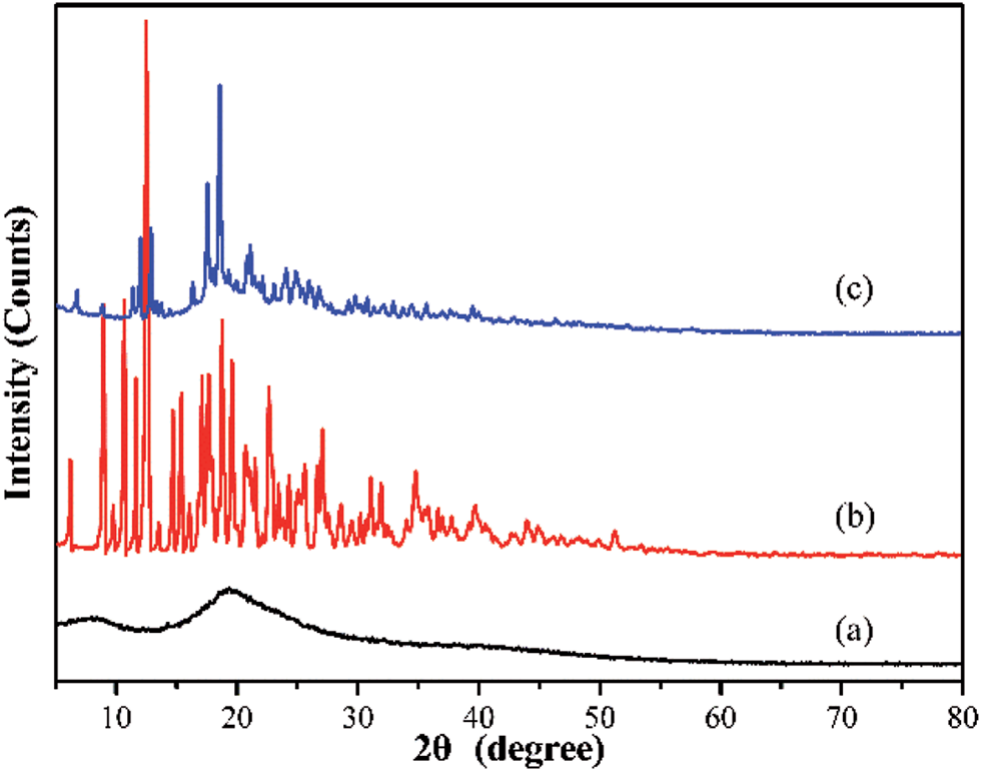
XRD patterns of BY (a), β-CD (b), and Y–β-CDP (c).

The XRD patterns of pristine BY, β-CD and Y–β-CDP at 2*θ*° are shown in Fig. 2. The pristine BY (Fig. 2a) has an obvious diffraction peak at about 2*θ* = 20° that corresponds to the amorphous phase of biomass and which is consistent with previous reports.^26^ The nine peaks in Fig. 2b, at 6.26, 8.91, 10.66, 12.51, 15.42, 17.13, 17.86, 18.78, and 19.53°, according to the JCPDS (Joint Committee on Powder Diffraction Standards) database (PDF no. 17-1024), belong to the characteristic peaks of β-CD. After modification, some characteristic peaks of β-CD appear on the spectrum of Y–β-CDP (Fig. 2c), suggesting that β-CD were successfully introduced into the BY surface. In addition, the data clearly reveals that the crystallinity of the Y–β-CDP polymer decreased due to the destruction of the intrinsic hydrogen bonds of BY and β-CD when β-CD was successfully grafted onto BY. As an important parameter, the crystallinity of Y–β-CDP will affect the accessibility between the internal adsorption sites and metal ions and plays a decisive role in the adsorption of metal ions.^27^ In other words, compared with β-CD, the decrease of the crystallinity of Y–β-CDP will be beneficial to enhance the adsorption capacity of metal ions.

### 3.2 Effect of the pH and pHz

**Fig. 3:**
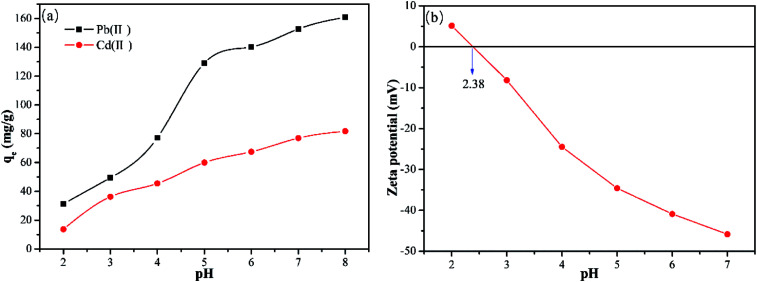
Effect of pH (a) on the adsorption of Pb(ii) and Cd(ii) onto Y–β-CDP (reaction conditions: Pb(ii) and Cd(ii) concentration of 50mg L^−1^ with Y–β-CDP dosage of 0.2g L^−1^ at 25 °C for 25 min) and (b) on zeta potential of Y–β-CDP.

It is necessary to examine the effect of the pH as it not only determines the surface charge of the adsorbent but also the form of heavy metal ions present.^28^ Thus, the effect of pH on the Y–β-CDP adsorption of Pb(ii) and Cd(ii) was evaluated by adjusting the pH values (2,3,4,5,6,7, and 8) of the solution. The data presented in Fig. 3a reveals that the adsorption capacity of metal ions adsorbed by Y–β-CDP was significantly affected by the pH of the solution. As the pH values increased from 2 to 8, the adsorption capacities of Pb(ii) and Cd(ii) increased from 31.35 to 160.9 mg g^−1^ and 13.75 to 81.75 mg g^−1^, respectively. This implies that the higher uptake capacity for Pb(ii) and Cd(ii) was achieved at higher pH values. The trend of the pH effect on removal of Pb(ii) and Cd(ii) was consistent with previous findings in the literature.^29–31^ This could be explained by the theory of isoelectric point, which was determined by measuring the zeta potential of the Y–β-CDP obtained at different pH values (see Fig. 3b).

The point of zero charge (pH_pzc_) of Y–β-CDP was 2.38. When pH < pH_pzc_, the surface of the Y–β-CDP was mainly positively charged. Thus, at low pH values, due to the influence of protonation and the presence of a large number of H^+^/H_3_O^+^ competing with metal ions for adsorption sites, the adsorption capacity was low. When pH > pH_pzc_, the increase in adsorption capacity is not obvious, which can be attributed to the following two reasons: (1) too high H^+^ concentration (low pH) of the solution is not good for Pb(ii)/Cd(ii) to replace H^+^ from –OH/–SH, and the positive direction of reaction would be limited more seriously with the decrease in pH. (2) Lower pH is beneficial to desorption to some extent. By increasing the pH value, the adsorption capacity was increased owing to the enhancement of the deprotonation effect and the negatively charged surface of Y–β-CDP, which strengthened the electrostatic attraction of metal ions and Y–β-CDP.^32^ Moreover, it is worth noting that the isolated electron pairs on the nitrogen and sulfur atoms of Y–β-CDP will coordinate with metal ions to form stable complexes and contribute to their uptake.^33^ Generally, the removal mechanism of Pb(ii) and Cd(ii) at low pH mainly involves ion exchange and at higher pH, the electrostatic attraction, coordination bond and complexation/chelation can be the possible mechanism.

Although the increase of pH will increase the adsorption capacity of metal ions, a precipitate of metal ions and hydroxide ions will be formed.^34^ Therefore, in order to avoid the interference of precipitation, the pH values of 5 and 7 were selected for removal of Pb(ii) and Cd(ii), respectively, in the subsequent experiments.

### 3.3 Effect of the adsorbent dosage

**Fig. 4:**
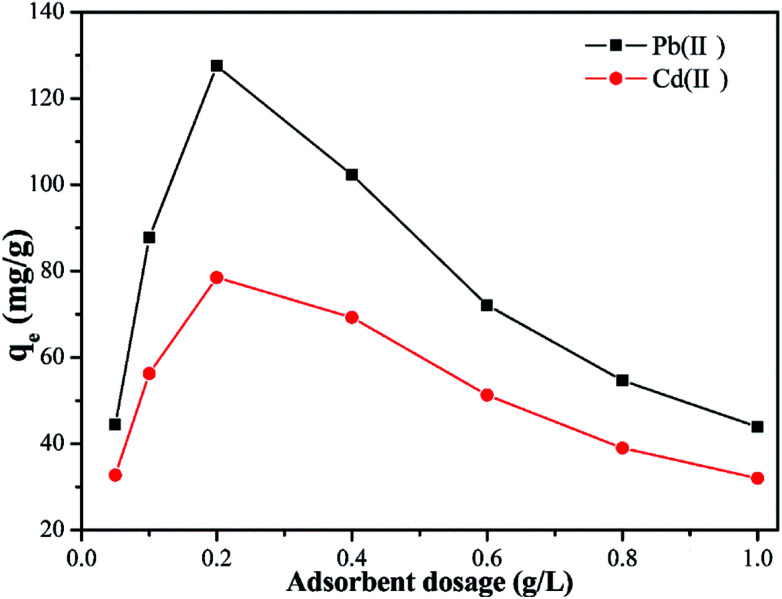
Effect of Y–β-CDP dosage on the adsorption of Pb(ii) and Cd(ii) (reaction conditions: Pb(ii) and Cd(ii) concentration of 50mg L^−1^ at 25 °C for 25 min).

The effect of Y–β-CDP dosage (0.05–1 g L^−1^) on the adsorption capacity of Pb(ii) and Cd(ii) was evaluated and the results of this evaluation are shown in Fig. 4. This results reveal that the adsorption capacity of Pb(ii) and Cd(ii) initially increases sharply with the increase of the Y–β-CDP dosage from 0.05 to 0.2 g L^−1^. This initial increase in the adsorption capacity promoted by the increase of the Y–β-CDP dosage resulted from the increase of the surface area and number of adsorption sites, which were completely occupied by a large number of metal ions in the aqueous solution. However, when the Y–β-CDP dosage rose from 0.2 to 1 g L^−1^, the Pb(ii) and Cd(ii) adsorption capacity decreased sharply. This may be due to the agglomeration effect brought about by the excessive amount of adsorbent and the discrepancy between the number of effective adsorption sites and insufficient metal ions in the solution,^35^ which ultimately lead to a failure to reach the adsorption equilibrium. Therefore, these results indicate that the 0.2 g L^−1^ dosage of Y–β-CDP was an adequate dosage to use in the subsequent experiments.

### 3.4 Effect of contact time and adsorption kinetics

**Fig. 5:**
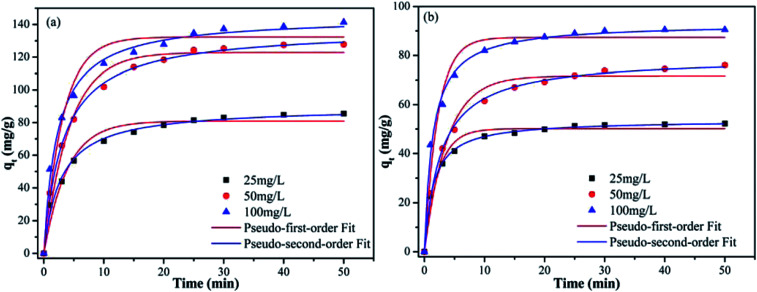
Adsorption kinetic curve of Pb(ii) (a) and Cd(ii) (b) on Y–β-CDP at different initial concentrations (reaction conditions: pH 5 for Pb(ii) and pH 7 for Cd(ii); Y–β-CDP dosage of 0.2 g L^−1^; 25 °C reaction temperature).

As shown in Fig. 5, the adsorption capacity of Pb(ii) and Cd(ii) on Y–β-CDP increased rapidly during the first 10 min because there were enough adsorption sites on the surface of Y–β-CDP. Afterward, however, it increased slowly and eventually reached equilibrium at around 25 min for both Pb(ii) and Cd(ii). This may be attributable to fewer available adsorption site on the surface of the adsorbent and the repulsive force that exist between metal ions adsorbed on Y–β-CDP and metal ions from the bulk solution.^3^ Accordingly, a 25 min contact time was selected for the subsequent adsorption experiments.

In addition, in order to elucidate the kinetic mechanism of Pb(ii) and Cd(ii) adsorption on Y–β-CDP, the pseudo-first-order and pseudo-second-order models were used to examine the experimental data, and their related nonlinear equations are given as eqn (3) and (4):^12^3
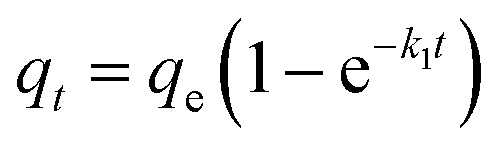
4
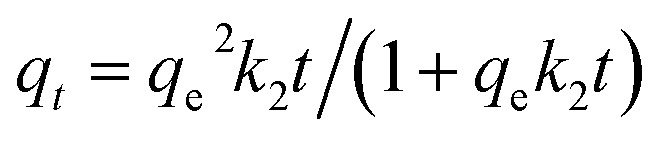
where *q*_*t*_ and *q*_e_ are the amount of adsorption at time *t* (min) and at equilibrium, respectively, both in mg g^−1^; *k*_1_ (min^−1^) and *k*_2_ (g (mg^−1^ min) are the adsorption rate constants.

The nonlinear fitting graphs and data on related parameters are presented in Fig. 5 and Table 1, respectively. These results reveal that, for different concentrations of metal ions, the correlation coefficient values (*R*^2^) obtained from pseudo-second-order models were 0.9929–0.9979 and 0.9950–0.9993 for Pb(ii) and Cd(ii), respectively, which are evidently higher than the 0.9524–0.9701 and 0.9548–0.9665 values of the correlation coefficients of the pseudo-first-order models. In addition, the predicted *q*_e_ values by the pseudo-second-order kinetic model are in good accordance with the experimental values. In contrast, the calculated *q*_e_ values of the pseudo-first-order are relatively distant from the experimental value. It can be deduced from these results that the pseudo-second-order adsorption mechanism plays a dominant role in the adsorption of Pb(ii) and Cd(ii) by Y–β-CDP and the chemisorption may be the rate-limiting step.^35^ In addition, the adsorption rate constant *k*_2_ was lower, which indicates that the binding sites on Y–β-CDP had higher affinity for Pb(ii) and Cd(ii) and were favorable for the adsorption process.

**Table 1:** Kinetic parameters for the adsorption of Pb(ii) and Cd(ii) onto Y–β-CDP

Metal ions	*C*_0_ (mg g^−1^)	Experimental (mg g^−1^)	Pseudo-first-order			Pseudo-second-order		
			*q*_e_ (mg g^−1^)	*k*_1_ (min^−1^)	*R* ^2^	*q*_e_ (mg g^−1^)	*k*_2_ (g (mg^−1^min))	*R* ^2^
Pb(ii)	25	88.25	80.90	0.258	0.9555	89.58	0.0040	0.9929
	50	134.05	122.91	0.232	0.9701	137.39	0.0023	0.9979
	100	143.5	132.28	0.313	0.9524	144.79	0.0031	0.9946
Cd(ii)	25	53.25	50.11	0.442	0.9665	53.69	0.013	0.9993
	50	79.75	71.67	0.270	0.9646	80.19	0.005	0.9979
	100	93	87.32	0.452	0.9548	95.20	0.008	0.9950

### 3.5 Effect of the concentration and adsorption isotherms

**Fig. 6:**
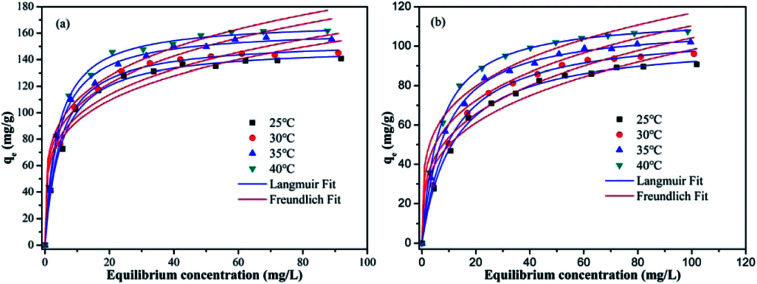
Adsorption isotherm curve of Pb(ii) (a) and Cd(ii) (b) on Y–β-CDP at different temperatures (reaction conditions: the initial concentration of Pb(ii) and Cd(ii) range from 10 to 120 mg L^−1^; pH 5 for Pb(ii) and pH 7 for Cd(ii), respectively; 0.2 g L^−1^ dosage of Y–β-CDP and 25 min of contact time).

To investigate the effect of the initial metal ions concentration and temperature on the adsorption capacity, the adsorption isotherms of Pb(ii) and Cd(ii) on Y–β-CDP were obtained at four different temperatures (25, 30, 35 and 40 °C) and are presented in Fig. 6. From these isotherms, it is obvious that the adsorption capacity was strengthened with the increase of the initial concentration of metal ions until equilibrium was reached. This can be explained by the increase of the possibility of contact between metal ions and adsorbent with the increase of the metal ion concentration, and high concentration of metal ions provides enough driving force to overcome mass transfer resistance.^36^ In addition, with the increase of the temperature, the adsorption equilibrium level increased gradually. This is because the increase of temperature increase the diffusion rate of Pb(ii) and Cd(ii) from the bulk solution to the adsorbent surface,^37,38^ and the adsorption of Pb(ii) and Cd(ii) on Y–β-CDP are endothermic reaction.

In this study, for purpose of explaining the removal mechanism, two commonly used isotherms equations, namely the Langmuir (eqn (5)) and Freundlich (eqn (6)) isotherms equations,^30^ were adopted to fit the equilibrium data and the corresponding isotherm constants are listed in Table 2.5
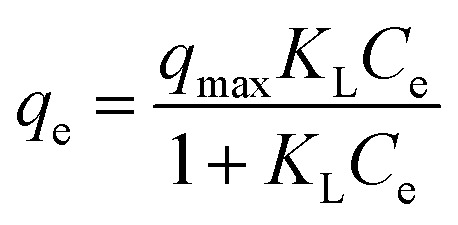
6
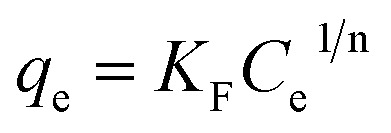
where *q*_max_ (mg g^−1^) represents the maximum adsorption capacity, *K*_L_ (L mg^−1^) is the adsorption constant, *C*_e_ (mg L^−1^) is the metal ions concentration at equilibrium, *K*_F_ (mg g^−1^) and *n* are the Freundlich constants related to adsorption capacity and adsorption intensity, respectively.

**Table 2:** Isotherm parameters for the adsorption of Pb(ii) and Cd(ii) onto Y–β-CDP

Metal ions	Temperature (°C)	Experimental (mg g^−1^)	Langmuir model			Freundlich model		
			*q*_max_ (mg g^−1^)	*K*_L_ (L mg^−1^)	*R* ^2^	*K*_F_ (mg g^−1^)	*n*	*R* ^2^
Pb(ii)	25	140.75	150.08	0.203	0.9961	55.54	4.426	0.9352
	30	145	154.59	0.216	0.9988	57.88	4.442	0.9398
	35	155.35	162.99	0.247	0.9936	62.12	4.437	0.9448
	40	161.7	168.97	0.261	0.9980	65.80	4.512	0.9452
Cd(ii)	25	90.6	102.80	0.085	0.9978	25.18	3.386	0.9583
	30	96	107.77	0.093	0.9992	27.67	3.475	0.9604
	35	102	111.00	0.120	0.9986	33.10	3.826	0.9627
	40	107.4	115.17	0.153	0.9996	39.14	4.215	0.9568

As shown in Table 2, compared with the results obtained with the Freundlich model, the adsorption process of Pb(ii) and Cd(ii) on Y–β-CDP are better described by Langmuir model according to the *R*^2^ values. The predicted *q*_max_ of Pb(ii) and Cd(ii) by Y–β-CDP according to the Langmuir model were consistent with the equalized maximum saturated adsorption capacity obtained from the experimental values. Thus, it can be concluded that Pb(ii) and Cd(ii) adsorption onto Y–β-CDP was dominated by monolayer adsorption processes. Additionally, the *K*_L_ value obtained from the Langmuir isotherm increases with the increase of the temperature. Moreover, it is also indicated that the adsorption process of Pb(ii) and Cd(ii) on Y–β-CDP is a chemical endothermic reaction, which is consistent with the experimental results. As to the Freundlich model, the calculated values of *n* were greater than 1, implying that the adsorption process was a favorable process and there is likely to be a high affinity between the adsorbate and the adsorbent.

More importantly, the maximum adsorption capacities of Pb(ii) and Cd(ii) obtained from the Langmuir isotherm at 25 °C were 150.08 and 102.80 mg g^−1^ when the initial concentration of metal ions was 120 mg L^−1^. Meanwhile, at 40 °C, the maximum adsorption capacities were 168.97 and 115.17 mg g^−1^, which is higher than that of various adsorbents reported in the literature (Table 3). These findings results reveal that Y–β-CDP has great competitiveness in the removal of heavy metal ions from contaminated water.

**Table 3:** Comparison of the Y–β-CDP adsorption capacity by various adsorbents

Adsorbents	Adsorption capacity (mg g^−1^)		Reference
	Pb(ii)	Cd(ii)	
Y–β-CDP	150.08	102.80	This study
Mesoporous activated carbon	20.3	27.3	3
EDTAD-modified magnetic baker's yeast	99.26	48.70	19
β-Cyclodextrin polymers	196.42	136.43	23
EDTA modified β-cyclodextrin/chitosan	114.8		24
Cystine-modified baker's yeast	45.87	11.63	39
Nano-ZnO/yeast composites	31.72	—	40
Ethylenediamine-modified yeast biomass	121.26	—	41
Poly(methacrylic acid) modified baker's yeast	243.9	108.7	43

### 3.6 Effect of temperature and thermodynamics analysis

**Fig. 7:**
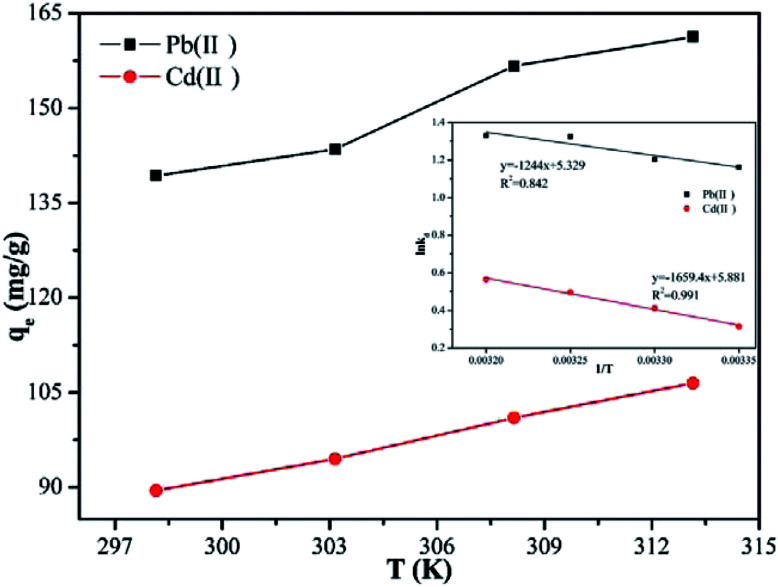
Effect of temperature on the adsorption capacity. Thermodynamics plot for adsorption of Pb(ii) and Cd(ii) by Y–β-CDP is shown as an inset. (reaction conditions: metal ions concentration is 120 mg L^−1^, pH 5 for Pb(ii) and pH 7 for Cd(ii), contact time is 25 min and 0.02 g Y–β-CDP in 100 ml solution).

Temperature is usually considered as an important factor in the adsorption process. Accordingly, it was deemed necessary to examine the effect of the temperature on the adsorption capacity. The results, which are shown in Fig. 7, reveal that, when the temperature was increased from 298.15 to 313.15 K, the adsorption capacity of Pb(ii) and Cd(ii) increased from 139.3 to 161.3 mg g^−1^, and from 89.5 to 106.5 mg g^−1^, respectively. This findings can be explained by two reasons. First, the collision efficiency between the heavy metal ions and the adsorbents was increased with increase of the temperature.^40^ Second, with higher ambient temperature, the deprotonation of the adsorption functional groups is enhanced, thus providing more adsorption sites to bind metal ions.^41^ Other materials have also been reported to show similar adsorption behavior of Pb(ii) and Cd(ii).

In order to further study the influence of the temperature on the adsorption capacity and elucidate the possible adsorption mechanism of Pb(ii) and Cd(ii) on Y–β-CDP, the relevant thermodynamic parameters for Pb(ii) and Cd(ii) adsorption were calculated. The standard Gibbs free energy change (Δ*G*), standard enthalpy change (Δ*H*) and standard entropy change (Δ*S*) were determined by using eqn (7) and (8):^10^7
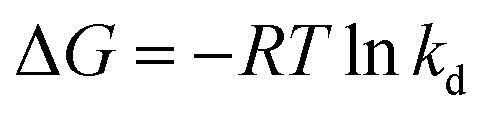
8
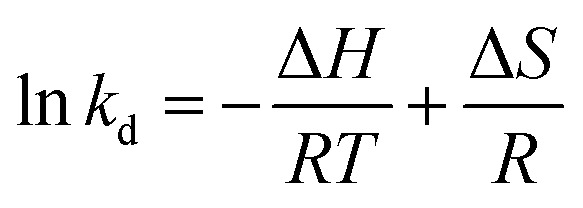
where *R* is the ideal gas constant (J mol^−1^ K^−1^), *T* is the temperature in Kelvin (K), and *k*_d_ is the Langmuir constant (L mol^−1^). The values of Δ*H* and Δ*S* can be calculated from the slope and intercept, respectively.

As shown in Table 4, the negative values of Δ*G* for Pb(ii) and Cd(ii) at all temperatures revealed that the adsorption process was essentially spontaneous. In addition, the *K*_d_ and absolute values of the ΔG gradually increases with the increase of temperature, suggesting that the degree of spontaneity increased with the rise of the temperature, which depends mainly on the chemical adsorption rather than physical adsorption.^42^ Moreover, the positive value of the Δ*H* further indicates that the adsorption processes were endothermic, which is consistent with the results that the adsorption capacity of Pb(ii) and Cd(ii) by Y–β-CDP increased with the increase of the temperature. The randomness at the solid–solution interface increases during the adsorption of Pb(ii) and Cd(ii) on Y–β-CDP,^40^ resulting in a positive value for the Δ*S*, and also suggests that the functional groups on Y–β-CDP has strong affinity for Pb(ii) and Cd(ii).

**Table 4:** Thermodynamic parameters for Pb(ii) and Cd(ii) adsorption

Metal ions	Temperature (°C)	*K*_d_ (cm^3^ g^−1^)	Δ*G* (kJ mol^−1^)	Δ*H* (kJ mol^−1^)	Δ*S* (J mol^−1^ K^−1^)
Pb(ii)	25	3.197	−2.881	10.343	44.31
	30	3.335	−3.036		
	35	3.761	−3.394		
	40	3.779	−3.461		
Cd(ii)	25	1.369	−0.779	13.796	48.89
	30	1.510	−1.039		
	35	1.640	−1.268		
	40	1.757	−1.467		

### 3.7 Regeneration of Y–β-CDP

**Fig. 8:**
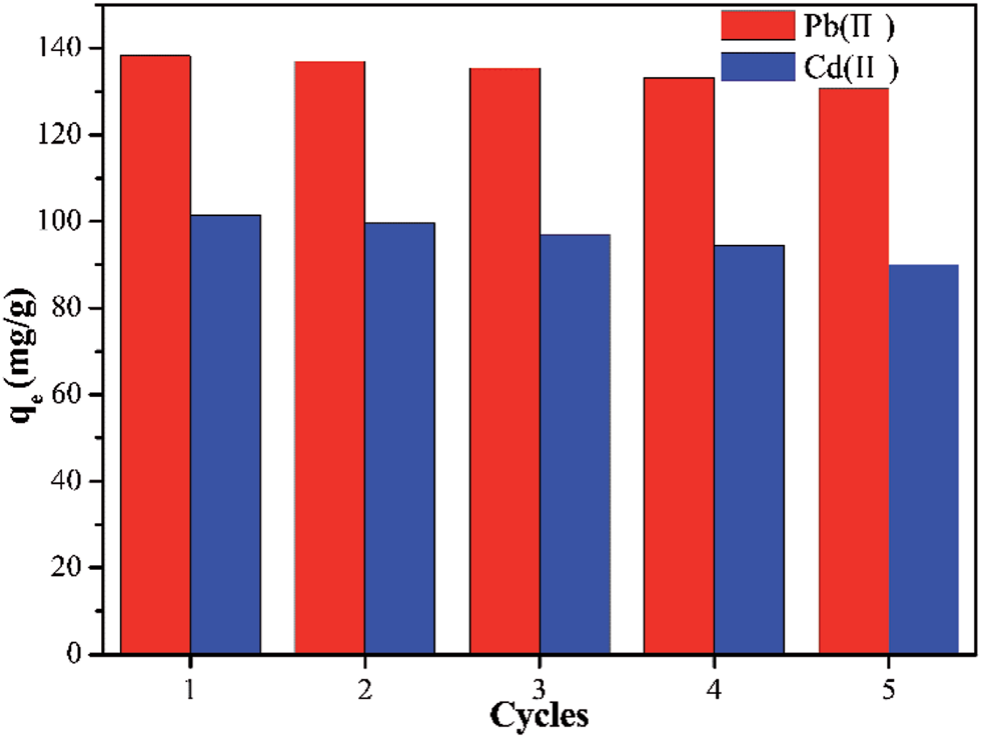
Adsorption capacity of Pb(ii) and Cd(ii) onto Y–β-CDP with five adsorption–desorption cycles.

An adsorbent with high adsorption properties and excellent recyclability will be more economical and competitive. In order to determine the regenerative performance of Y–β-CDP, five continuous adsorption–desorption cycles were performed using a 0.1 M HCl solution to elute the saturated adsorbent. The results shown in Fig. 8 reveal that the adsorption capacity of the regenerated Y–β-CDP was slightly reduced with the increase number of cycles, and the adsorption capacity decreased from 138.2 to 130.6 mg g^−1^ for Pb(ii) and from 101.5 to 90 mg g^−1^ for Cd(ii). Regarding the results, what is worth paying attention to is that although the adsorption capacity decreased gradually, it still remained at a satisfactory level. Thus, the adsorption–desorption experiment results confirmed that Y–β-CDP can be a renewable and competitive adsorbent for Pb(ii) and Cd(ii) adsorption.

### 3.8 Adsorption mechanisms

In order to investigate the adsorption mechanism, the Y–β-CDP were characterized by FT-IR spectroscopy and XPS analyses before and after metal ions adsorption.

#### 3.8.1 FTIR spectroscopy analysis

**Fig. 9:**
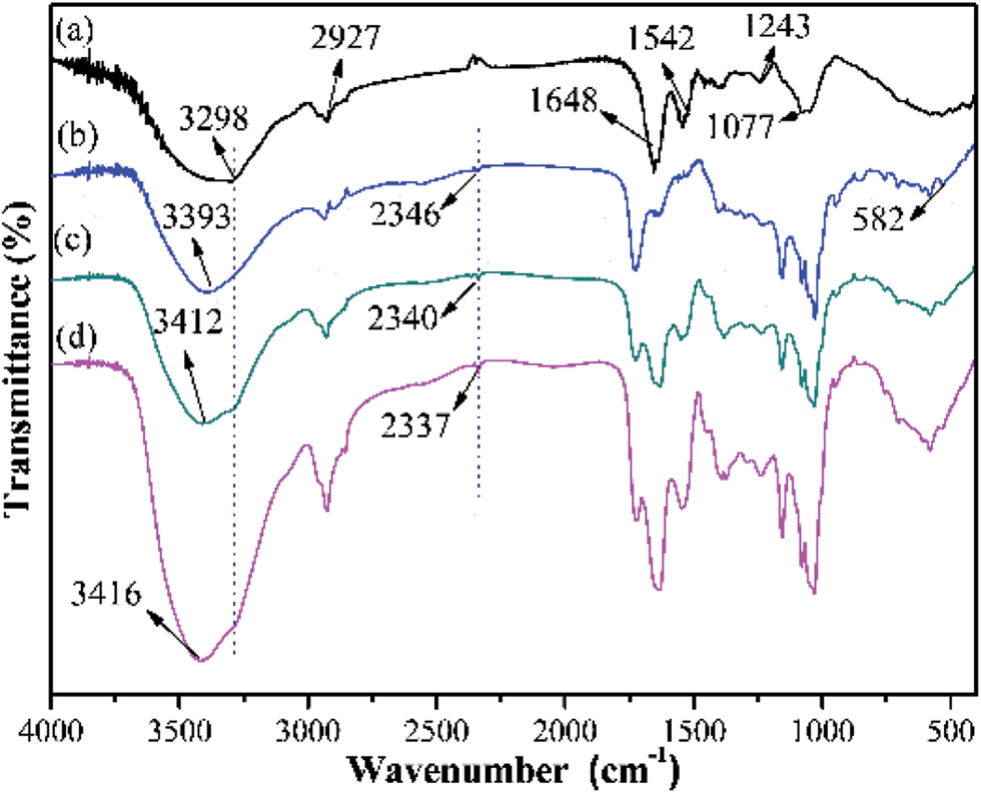
FTIR spectra of BY (a), Y–β-CDP (b), Y–β-CDP–Pb(ii) (c) and Y–β-CDP–Cd(ii) (d).

FTIR spectroscopy has been an effective method to distinguish specific functional groups in a molecule, as each contains a certain chemical bond that usually exhibits a unique adsorption band.^42^ As shown in Fig. 9a, peaks at 3298, 2927, 1648, 1542, 1243 and 1077 cm^−1^ are present in the BY spectrum.^43^ Typically, the strong and broad band at around 3298 cm^−1^ is attributed to the overlapping O–H and N–H stretching bands, while the peak at 2927 cm^−1^ corresponds to the C–H stretching vibrations of CH, –CH_2_ and CH_3_ groups.^44,45^ The peaks at 1648 and 1542 cm^−1^ conform with those of the C=O and N–H stretching vibration, and the peaks at 1243 and 1077 cm^−1^ can be assigned to the C–N and C–O stretching vibration, respectively.^46^ In the Y–β-CDP polymer (Fig. 9b), the peak at 3298 cm^−1^ shifted to 3393 cm^−1^, and the area of this peak was broadened. In addition, the new peaks located at 2346 cm^−1^ and 582 cm^−1^ are characteristic of –SH groups.^47^ These results indicate that thiomalic acid and polyhydroxy β-CD have been introduced on the BY surface and the Y–β-CDP has been prepared successfully. After Y–β-CDP loaded Pb(ii) and Cd(ii) (Fig. 9c and d), the peak at 3393 cm^−1^ was clearly shifted to 3412 cm^−1^ for Pb(ii) and to 3416 cm^−1^ for Cd(ii). This is probably due to the –OH and –NH groups present in Y–β-CDP which definitely participate in the adsorption of metal ions. In addition, the peaks of –SH group shifted from 2346 to 2340 cm^−1^ for Pb(ii) and to 2337 cm^−1^ for Cd(ii), which also demonstrate that the –SH groups are involved in the adsorption of Pb(ii) and Cd(ii). Similar spectral changes after adsorption of metal ions have been reported by using NH_2_-functionalized ZrMOFs,^10^ nano-ZnO/yeast composites^39^ and Fe_3_O_4_-CS-L^48^ as adsorbent.

#### 3.8.2 XPS analysis

The kinetic and isotherm results showed that the chemisorption of the monolayer on the surface of Y–β-CDP plays a major role in the removal of Pb(ii) and Cd(ii).

**Fig. 10:**
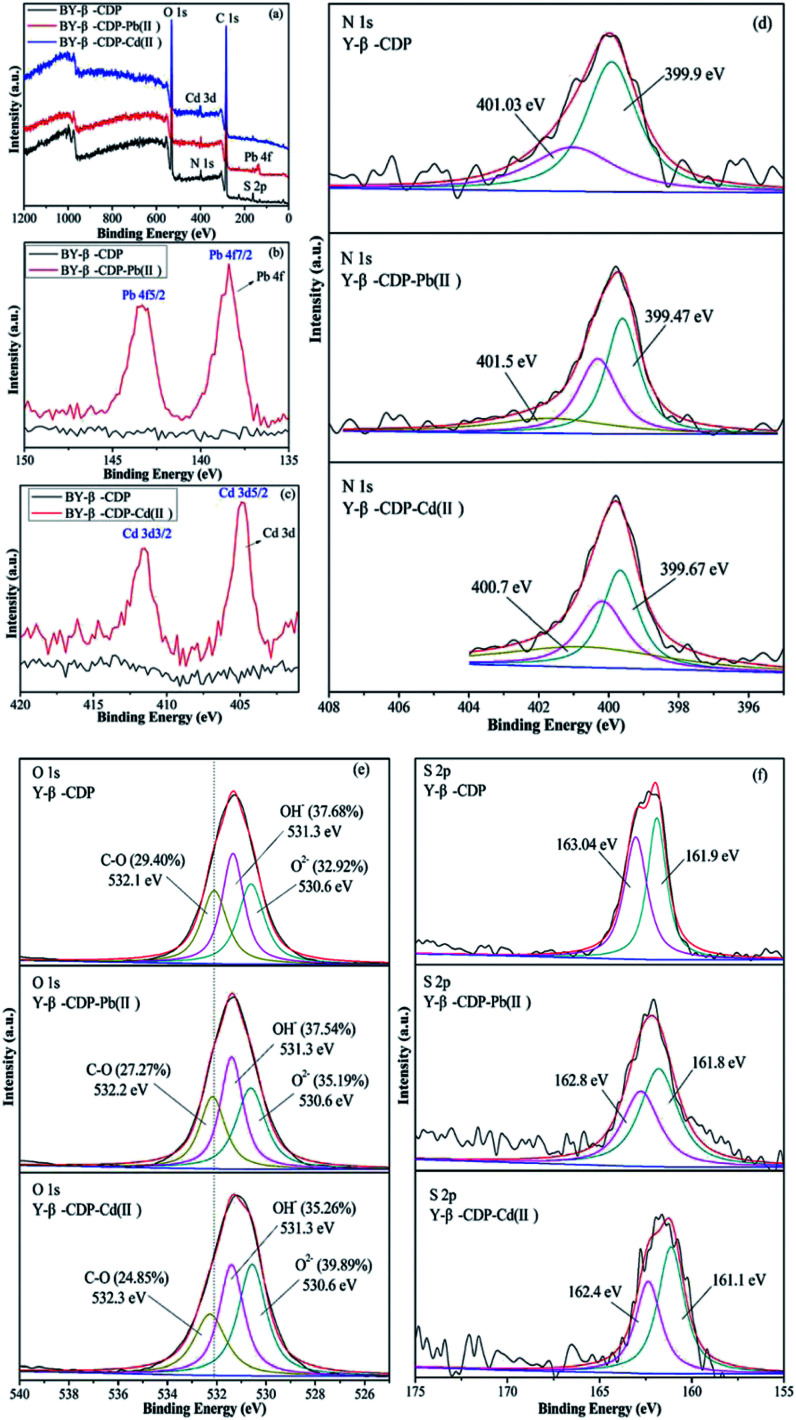
XPS of Y–β-CDP before and after adsorption for metal ions: (a) broad scan, (b) Pb 4f, (c) Cd 3d, (d)N 1s, (e) O 1s, and (f) S 2p.

To further investigate the interactions and establish the adsorption mechanisms between metal ions and Y–β-CDP, XPS analysis of the Y–β-CDP surface was performed before and after metal ions adsorption. The broad XPS spectra of Y–β-CDP and Pb(ii) or Cd(ii) absorbed on Y–β-CDP are displayed in Fig. 10a. The spectra show that the chemical components of Y–β-CDP are C, N, O, and S, which is consistent with the results of the EDS analysis. The high resolution XPS spectra of the Pb 4f and Cd 3d are shown in Fig. 10b and c after Pb(ii) and Cd(ii) adsorption, respectively. The stronger peaks at 138.4 and 143.3 eV were ascribed to Pb 4f7/2 and Pb 4f5/2,^49^ similar conspicuous peaks at 404.8 and 411.5 eV were assigned to Cd 3d5/2 and Cd 3d3/2,^50^ respectively. These findings indicate that the Y–β-CDP can effectively capture metal ions from aqueous solution.

It is generally recognized that the main reason for the adsorption of metal ions is the chemical interaction between the metal ions and adsorbent. Thus, in order to study the effects of N, O and S functional groups on Pb(ii) and Cd(ii) adsorption, the narrow spectra of N 1s, O 1s, and S 2p are shown in Fig. 10d–f. The N 1s peak fitting results of Y–β-CDP showed two peaks at 399.9 and 401.03 eV. The peak at 399.9 eV corresponds to –NH_2_, which is located on the structural unit of BY. The second peak at 401.03 eV was attributed to –NH_3_^+^ (amino-protonated).^51,52^ After Pb(ii) adsorption, the peak at 399.9 eV of Y–β-CDP shifted to 399.47 eV, and it is worth noting that there is a higher new peak emerged at 401.5 eV. Similarly, after Cd(ii) adsorption, the peak at 399.9 eV of Y–β-CDP shifted to 399.67 eV and a higher new peak appeared at 400.7 eV. These findings indicate that the –NH–Pb(ii)/Cd(ii) complexes were formed after Y–β-CDP adsorbed Pb(ii) and Cd(ii), and a lone pair of electrons that exist in the nitrogen atom can form a stable coordination bond with metal ions, while the density of the electron cloud around the nitrogen atoms decreases and the binding energy increases.^53,54^ These findings indicate that N atoms are involved in metal ions adsorption.

**Fig. 11:**
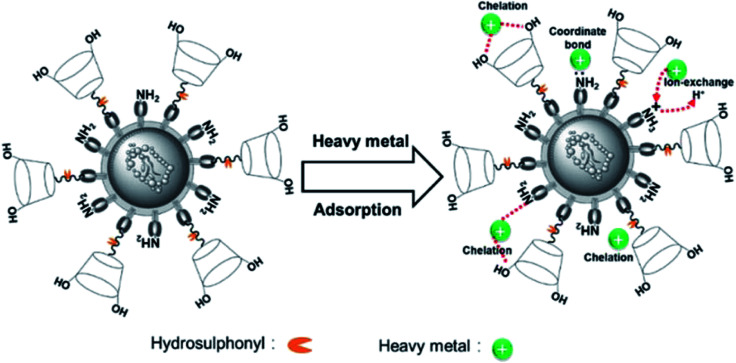
The probable mechanisms of Pb(ii) and Cd(ii) adsorption on Y–β-CDP.

The O 1s spectra (Fig. 10e) was displayed three peaks located at 530.6, 531.3, and 532.1 eV, which were attributed to metal oxide (O–M), hydroxyl bonded to metal (OH–M), and the carbon–oxygen single bond (C–O), respectively.^53,55,56^ Before adsorption, the area ratio of O–M, OH–M, and C–O were 32.92, 37.68, and 29.40%, and after Pb(ii) adsorption, their area ratio changed to 35.19, 37.54, and 27.27%. Additionally, after Cd(ii) adsorption, their area ratio changed to 39.89, 35.26, and 24.85%. Thus, it is obvious that the area ratio of OH-M decreased after metal ions adsorption, whereas the area ratio of O–M increased due to the formation of O–M^2+^ groups on the Y–β-CDP surface. In addition, the binding energy of Y–β-CDP at 532.1 eV increased slightly after Y–β-CDP adsorbed metal ions, which indicates that the hydroxyl groups in the β-CD structure may be involved in weak interaction with the adjacent amino groups to remove metal ions together.^24,57^ The spectrum of S 2p (Fig. 10f) before adsorption displays two peaks at 161.9 and 163.04 eV, which correspond to the –SH.^58^ After Y–β-CDP loaded metal ions, the peak at 163.04 eV shifted to 162.8 eV for Pb(ii) and 162.4 eV for Cd(ii). It is generally recognized that the chemical shifts should be considered when the binding energy clearly changes (>0.5 eV).^59^ These results indicate that sulfhydryl groups on thiomalic acid also play an important role in removing metal ions. The interpretation of the FTIR spectroscopy and XPS analyses revealed that the adsorption of Pb(ii) and Cd(ii) on Y–β-CDP mainly depends on ions exchange, chelation, and coordination. The probable mechanisms of Pb(ii) and Cd(ii) adsorption on Y–β-CDP are shown in Fig. 11.

## 4. Conclusion

The objectives of this work were to determine the removal performance of Pb(ii) and Cd(ii) from aqueous solution by β-cyclodextrin grafted onto baker's yeast (Y–β-CDP) and elucidate the removal mechanisms involved. The successful preparation of the Y–β-CDP, with a low-cost and feasible simple method, was confirmed by FTIR spectroscopy, SEM and EDS analysis. The results showed that Y–β-CDP has excellent adsorption properties for Pb(ii) and Cd(ii). The adsorption of Pb(ii) and Cd(ii) on Y–β-CDP reached equilibrium in 25 min, and the kinetic process conformed to the pseudo-second order model. The adsorption isotherm data were well-fitted by the Langmuir model and the maximum adsorption capacity was 150.08 and 102.80 mg g^−1^ for Pb(ii) and Cd(ii), respectively, at 25 °C. The adsorption of metal ions on Y–β-CDP was demonstrated to be a spontaneous and endothermic process. Also, Y–β-CDP exhibited an excellent adsorption property for metal ions even after six adsorption–desorption cycles. According to adsorption characteristics and the result of the FTIR spectroscopy, XPS analysis, the adsorption mechanisms involved ions exchange, chelation, and the formation of coordinate bond with metal ions by the isolated electron pair at the N atoms. This study demonstrated that the Y–β-CDP can act as an effective adsorbent in the removal of metal ions from contaminated water.

## Conflicts of interest

There are no conflicts to declare.

## Supplementary Material
